# Unsupervised Data-Driven Reconstruction of Molecular Motifs in Simple to Complex Dynamic Micelles

**DOI:** 10.1021/acs.jpcb.2c08726

**Published:** 2023-03-09

**Authors:** Annalisa Cardellini, Martina Crippa, Chiara Lionello, Syed Pavel Afrose, Dibyendu Das, Giovanni M. Pavan

**Affiliations:** † Department of Innovative Technologies, University of Applied Sciences and Arts of Southern Switzerland, Polo Universitario Lugano, Campus Est, Via la Santa 1, 6962 Lugano-Viganello, Switzerland; ‡ Department of Applied Science and Technology, Politecnico di Torino, Corso Duca degli Abruzzi 24, 10129 Torino, Italy; ¶ Department of Chemical Sciences and Centre for Advanced Functional Materials, Indian Institute of Science Education and Research (IISER) Kolkata, Mohanpur 741246, India

## Abstract

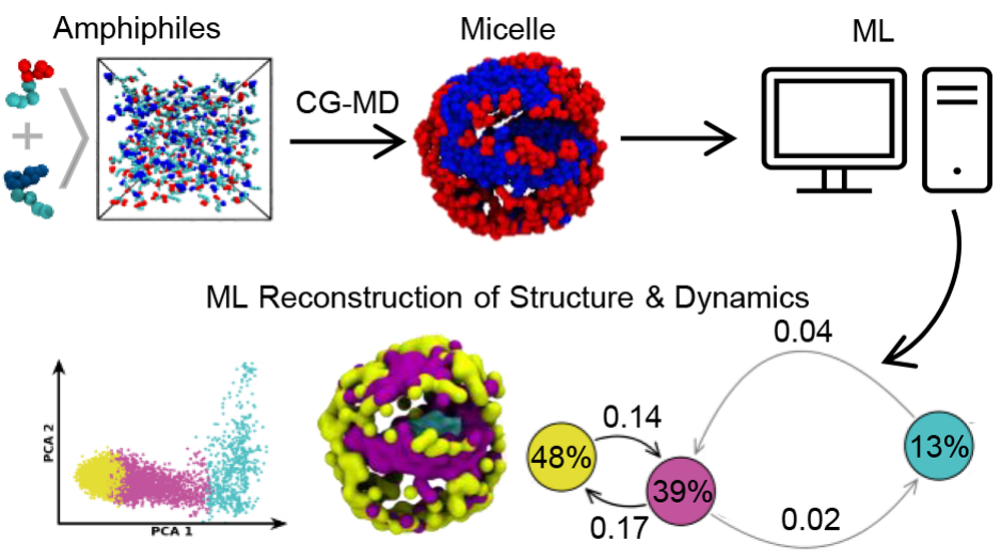

The reshuffling mobility of molecular building blocks in self-assembled micelles is a key determinant of many their interesting properties, from emerging morphologies and surface compartmentalization, to dynamic reconfigurability and stimuli-responsiveness. However, the microscopic details of such complex structural dynamics are typically nontrivial to elucidate, especially in multicomponent assemblies. Here we show a machine-learning approach that allows us to reconstruct the structural and dynamic complexity of mono- and bicomponent surfactant micelles from high-dimensional data extracted from equilibrium molecular dynamics simulations. Unsupervised clustering of smooth overlap of atomic position (SOAP) data enables us to identify, in a set of multicomponent surfactant micelles, the dominant local molecular environments that emerge within them and to retrace their dynamics, in terms of exchange probabilities and transition pathways of the constituent building blocks. Tested on a variety of micelles differing in size and in the chemical nature of the constitutive self-assembling units, this approach effectively recognizes the molecular motifs populating them in an exquisitely agnostic and unsupervised way, and allows correlating them to their composition in terms of constitutive surfactant species.

## Introduction

Understanding the structural environments which characterize soft supramolecular assemblies and their intrinsic dynamics is of prime importance toward the rational design of self-assembling materials with controllable dynamic properties.^1−5^ Recent studies suggest that the mobility of building blocks and the formation of dynamically diverse domains within the assemblies may significantly impact their properties, in a similar way as they do in natural systems.^3,6,7^ This is the case, for example, of supramolecular polymers where the intrinsic reshuffling dynamics of their monomers occurring on defected domains controls directly how fastly/slowly the polymeric fiber can reorganize its structure in response to a specific stimulus.^8^

In general, the possibility to create a certain degree of disorder (i.e., defects) in soft supramolecular assemblies is key for tuning and modulating fluid-like domains,^9,10^ controlling their stability,^11^ triggering stimuli-responsive attitude,^12^ and speeding-up chemical reactions.^13,14^ For instance, the relative mobility of lipids with temperature variations is at the origin of gel-to-liquid phase transitions in lipid bilayers,^10^ which has a clear impact first on the dynamics of membrane receptors and proteins embedded within them and secondarily on the complex functionalities that such a mobility controls, like the formation of rafts on cells surface.^15,16^ As another example, detailed comprehension of Lipid Liquid Nanoparticles (LLNs) and specifically of the fluidity of lipid building blocks within them, is crucial in developing stable drug delivery vehicles.^11^ Multicomponent assemblies are also attracting considerable interest thanks to the possibility of attaining complex dynamic functions.^12,17^ In particular, multicomponent surfactant micellar-like assemblies have been used as platforms to obtain dissipative supramolecular systems with “living” character or supramolecular reactors with interesting catalytic properties.^13,14,18^

Despite the massive experimental works to study structural properties (e.g., via TEM, AFM, SAXS, DLS, UV–vis, CD spectroscopy, etc.),^4,5,7,19−21^ unveiling the intrinsic dynamics of such soft disordered supramolecular materials still present daunting challenges. Experimental techniques based on, e.g., Förster Resonance Energy Transfer (FRET),^21^ Stochastic Optical Reconstruction Microscopy (STORM),^22^ or hydrogen/deuterium exchange (HDX) mass spectrometry,^23^ to name a few, permit us to elucidate the native behavior of the assemblies at the level of statistical ensembles, exploring dis-homogeneities, rearrangements, and conformational states with a resolution of 20–50 nm.^21,22,24−27^ However, the comprehension of the intrinsic mobility in complex soft assemblies requires studying it at a submolecular resolution, which is ambitious experimentally.

In this context, all-atom (AA) and coarse-grained (CG) molecular dynamics (MD) simulations are demonstrating a remarkable potential for the characterization of soft self-assembled materials. The high resolution and flexibility of the models enable us to explore molecular reshuffling processes within the assemblies^3^ and, more recently, also among them, thereby clarifying how the whole aggregates exchange molecular fragments and communicate with each other.^28^ Computational studies also facilitate the discover of the adaptability and stimuli-responsiveness of nanoparticles, micelles, or hydrogels, providing useful insights toward the rational design of controllable soft materials.^29,30^ Regardless of the numerous advantages, high-resolution molecular simulations provide a large amount of high-dimensional data, which are often nontrivial to analyze. Machine-learning (ML) approaches have demonstrated an impressive efficiency in identifying molecular motifs in self-organizing structures, such as, e.g., defects in supramolecular polymer fibers^9,31^ or the phase coexistence in lipid bilayers simulations.^10^

Multicomponent soft assemblies are characterized by an intrinsically higher level of complexity which is extremely difficult to unravel.^32,33^ Typically, in such aggregates, diverse chemical groups of the constituent molecules are engaged in various specific and nonspecific interactions with each other and with the solvent.^34^ In this condition, predicting the structural rearrangement and internal reorganization of diverse functional groups with an arbitrary chemical composition is far from trivial. Moreover, even the most sophisticated classical molecular thermodynamics theories of aggregation may be insufficient to give a complete overview of complex multicomponent assemblies. In particular, how different building blocks arrange in the assembly, to what extent they intermix or segregate, and whether they are able to exchange/reshuffle after controlling specific key-factors are relevant aspects typically elusive to ascertain. MD simulations certainly provide a qualitative picture concerning the internal reshaping of aggregates; however, an unequivocal connection/relation between structural motives and chemical compounds is extremely challenging without a thorough analysis of the MD trajectories. In addition, traditional approaches to analyze such trajectories with system-specific descriptors can be rather labor-intensive, and weakly transferable.^35−37^

Here we design a multiscale modeling approach to reconstruct the structural and dynamic complexity of bicomponent surfactant micelles which are used as a representative case study of multicomponent dynamic assemblies. Unsupervised clustering analysis of high-dimensional Smooth Overlap of Atomic Position (SOAP) data extracted from equilibrium molecular dynamics (MD) trajectories provides the pathway both to identify the main clusters emerging on the micelles and to reconstruct their dynamic interconnection.

First, we use a minimalistic physical model to confirm the key parameters controlling the segregation/intermixing of different surfactants in the micelles. Such a design model of micelles provides a reliable benchmark to validate our ML-based analysis protocol. Then, finer, chemically relevant molecular models of realistic bicomponent surfactant micelles^13,14^ have offered the framework to understand to what extent such general features govern realistic bicomponent self-aggregates. We demonstrate how the unsupervised analysis presented here is effective to identify and distinguish a number of structural environments inside a bicomponent micelle, different in terms of molecule ordering and rearrangements, and to correlate them to the specific surfactant species. In general, we suggest a versatile platform and general-purpose insights, useful to understand and control the global and microscopic structural/dynamical features of complex multicomponent self-assembled materials.

## Methods

### Minimalistic Coarse-Grained (mCG) Model

**Figure 1:**
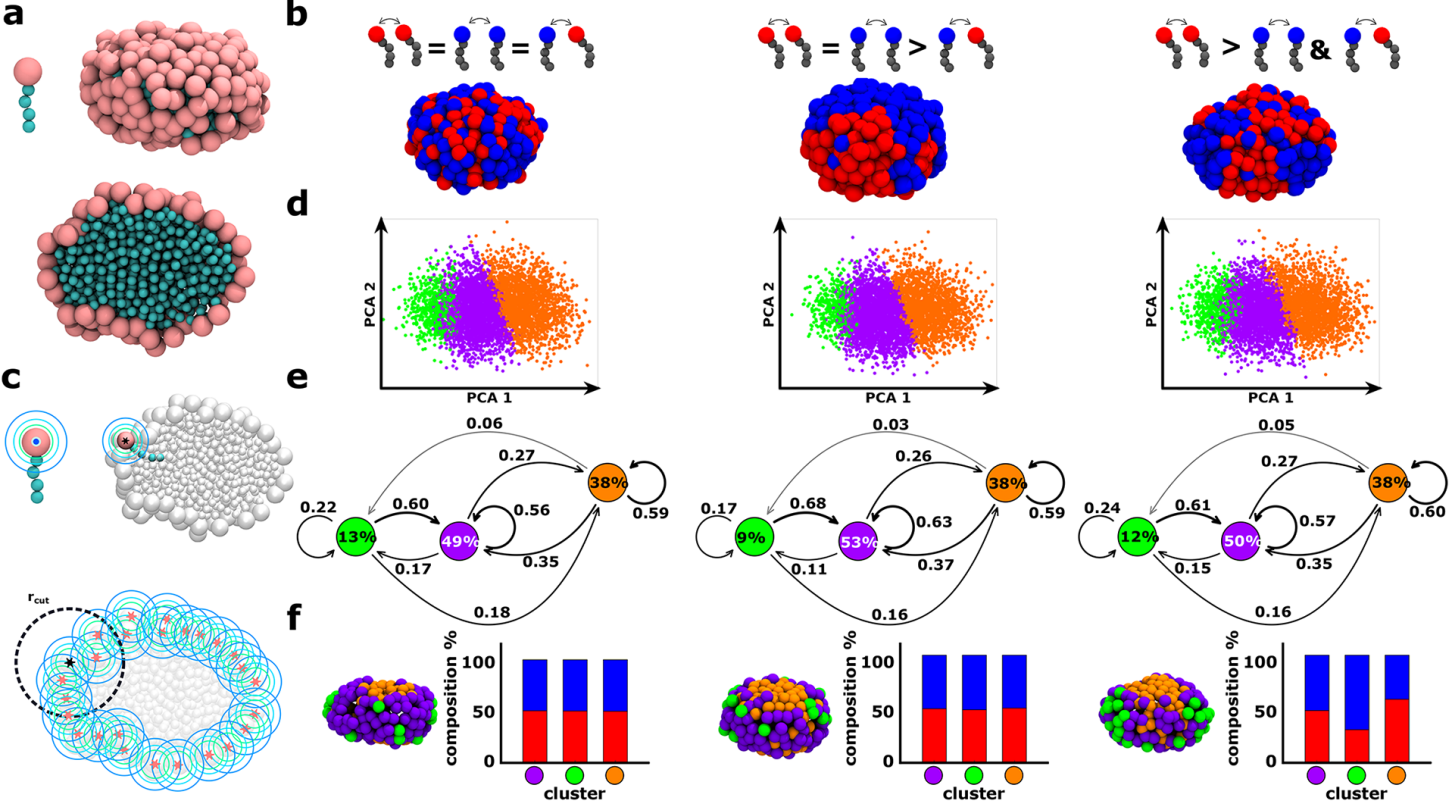
Minimalistic mCG models and unsupervised ML analysis of complex micelles with variable intersurfactant interactions. (a) Surfactant and micelle models (solvophilic surfactant head and solvophobic tail beads in pink and cyan, respectively). (b) Equilibrium configurations of three mCG micelles composed of 200 surfactants each. Every single micelle is characterized by two surfactant species, 100 red (**R**) plus 100 blue (**B**), respectively, with the same size of head beads (LJ parameters σ_**R**_ = σ_**B**_; see Methods for details). The mCG-MD simulations show that complete mixing is obtained with LJ ϵ_**RR**_ = ϵ_**BB**_ = ϵ_**RB**_. A net compartmentalization is observed for ϵ_**RR**_ = ϵ_**BB**_ > ϵ_**RB**_. An intermediate rearrangement is obtained with ϵ_**RR**_ > ϵ_**BB**_ and ϵ_**RR**_ > ϵ_**RB**_. (c) SOAP-based analysis scheme revealing the structural and dynamical features of the surfactant environments within a micelle. One SOAP vector is centered in each surfactant head monitoring its surrounding within a certain SOAP cutoff along the equilibrium MD trajectories. (d) Unsupervised clustering of SOAP data. Principal component analysis (PCA) of the SOAP data (projected on the first two PC components, PCA1 and PCA2). Three main clusters are identified via PAMM unsupervised clustering (green, purple, and orange) in all cases. (e) Interconversion diagrams showing the dynamics of surfactant transitions among the identified clusters. The percentages in the colored circles represent the equilibrium populations of the various detected clusters/states. The numbers on the transition arrows are the transition probabilities among the clusters in the time interval of the analysis, d*t*. (f) Left: Equilibrium snapshots of the various micelles (left-to-right) depicting the SOAP clusters distribution on their surface. Right: Composition of the SOAP clusters in terms of **R** and **B** amphiphiles for all cases.

**Figure 2:**
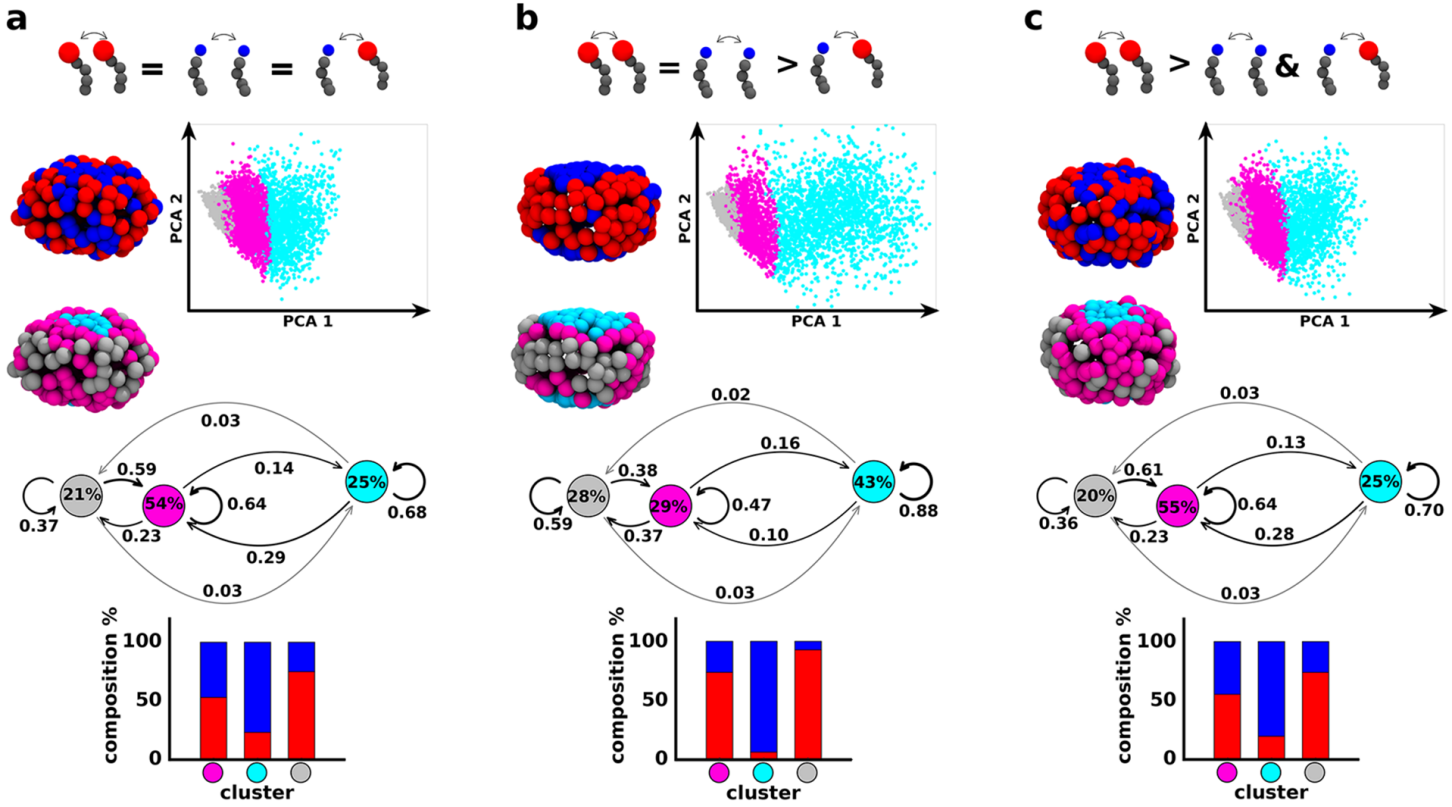
Minimalistic mCG models and unsupervised ML analysis of complex micelles with different surfactant head sizes and variable intersurfactant interactions. (a) Partial **R** and B amphiphile mixing is obtained for LJ ϵ_**RR**_ = ϵ_**BB**_ = ϵ_**RB**_. (b) A net compartmentalization of **R** and **B** is observed at ϵ_**RR**_ = ϵ_**BB**_ > ϵ_**RB**_. (c) An intermediate mixing/segregation is captured for ϵ_**RR**_ > ϵ_**BB**_ and ϵ_**RR**_ > ϵ_**RB**_. (a–c) Top: Equilibrium mCG-MD snapshots of various micelles made of **R** and **B** surfactants having LJ σ_**R**_/σ_**B**_ = 1.49 (left); projection of the SOAP-data set PCA on the first two Principal Components (right). Three SOAP clusters are identified: gray, magenta, and cyan. (a–c) Center: equilibrium mCG-MD micelle snapshots showing the SOAP clusters distribution and their dynamic interconversion diagrams (percentage cluster populations inside the colored circles, transition probabilities on the arrows connecting the various clusters). (a–c) Bottom: SOAP cluster compositions in terms of **R** and **B** amphiphiles.

The minimalistic CG model (mCG) is based on two types of elementary amphiphilic-like molecules, **R** and **B**, respectively (Figure 1c: identified with red and blue heads). **R** and **B** share common features. They are both composed of five beads each: one bead for the head (pink) and four beads modeling the tails (cyan) (Figure 1a, top). These five beads are bonded with harmonic potentials to form a linear structure, while their nonbonded interactions are described by Lennard-Jones (LJ) potentials. The mCG is an implicit-solvent model, in that there is no explicit treatment of the solvent molecules, but the head–head, head–tail, and tail–tail interactions are optimized to implicitly account for the presence of the solvent. In particular, all LJ parameters have been optimized in such a way to reproduce the typical behavior of surfactants self-assembling in a micelle: the hydrophobic tails pointing inside a shell of hydrophilic heads (see the Supporting Informaiton for topology and force field details). This was done via preliminary development of an explicit-solvent analogous mode, in the range of standard MARTINI force field parameters,^38^ where we simulated the self-assembly of **R** and **B** molecules in explicit polar solvent (see Figure S1 in the Supporting Information). The implicit-solvent mCG model was thus optimized to behave consistently with its explicit-solvent counterpart (see Figure S1 in the Supporting Information), while at the same time allowing an enhanced sampling of the surfactants reshuffling within the micelle. The models in Figure 1c have σ_**RR**_ = σ_**BB**_ = 0.7 nm, while those in Figure 2a–c, top, have σ_**RR**_ = 0.7 nm and a reduced σ_**BB**_ = 0.47 nm. The ϵ_**RB**_ determining the depth of the LJ interaction potential for the interspecies heterointeraction energy between **R** and **B** heads was kept constant in all mCG systems (ϵ_**RB**_ = 0.5 kJ mol^–1^), while the intraspecies homointeraction (i.e., ϵ_**RR**_ and ϵ_**BB**_) was varied to promote mixing (ϵ_**RR**_ = ϵ_**BB**_ = 0.5 kJ mol^–1^), segregation (ϵ_**RR**_ = ϵ_**BB**_ = 4 kJ mol^–1^), or an intermediate behavior (ϵ_**RR**_ = 4 kJ mol^–1^ and ϵ_**BB**_ = 0.5 kJ mol^–1^). Note that the features of tail beads were kept constant for all the case studies and are identical in both **R** and **B** molecules. Specifically, for each mCG tail bead σ_*tail*_ = 0.47 nm, while ϵ_*tail*,*tail*_ = 5 kJ mol^–1^, defining both the intra- and interspecies interactions (see Tables S1 and S2 in the Supporting Information for a summary of the LJ parameters of the mCG models). Complete details of both molecular models and simulation parameters (input files, etc.) are available at https://zenodo.org/record/7696708#.ZAI0A3bMI2w.

#### mCG-MD Simulations

All CG-MD simulations of the minimalistic model were carried out using the GROMACS software^39^ (versions 2018.6 and 2020.2) and have been performed in *NVT* conditions (constant *N*, number of particles, *V*, volume, *T*, temperature, during the MD runs). The volume of the simulation box was set to 20 × 20 × 20 nm^3^, and the simulations have been conducted in periodic boundary conditions. In all simulations, the temperature was kept at *T* = 300 K. In all mCG micelle models, the number of molecules is *N*_**R**_ = 100 and *N*_**B**_ = 100 surfactant molecules.

After a short preliminary minimization/relaxation, the MD runs were performed in implicit-solvent via Langevin dynamics using the stochastic dynamics (sd) integrator, where the parameter tau-t = 0.1 ps accounts for both the friction of the solvent and thermal fluctuations of the system. The time step was set at *Δt* = 40 fs, and the nonbonded interaction potentials were truncated and shifted at *r*_*c*_ = 1.2 nm. For each self-assembly simulation, we performed at least 20 μs of CG-MD, sampling the conformations every 1 ns. We started from randomly dispersed monomers, and we kept for the analysis just the equilibrium part of each trajectory, i.e., the last 5 μs. Regarding the mixed system (**RR** = **BB** = **RB**, in Figure 1b), we performed a longer simulation of 40 μs, keeping always the last 5 μs for the analysis, as representative of the equilibrium of the system (see also Figure S2 in the Supporting Information).

The CG-MD simulations of the control model in explicit solvent were performed in *NPT* conditions (constant *N*, number of particles, *P*, pressure, *T*, temperature, during the MD run), using the md integrator, with a time step of *Δt* = 40 fs. The equilibrated part of the simulations is 5 μs long, and the conformations have been sampled every 1 ns. The temperature of the system was kept constant using the velocity rescaling (v-rescale) thermostat,^40^ with time constant τ_*T*_ = 1 ps and coupling temperature *T* = 300 K. The pressure was also kept constant by the Parrinello–Rahman barostat,^41^ with time constant τ_*p*_ = 8 ps and reference pressure *p* = 1 bar.

### All-Atom (AA) and Finer Coarse-Grained (fCG) Models

**Figure 3:**
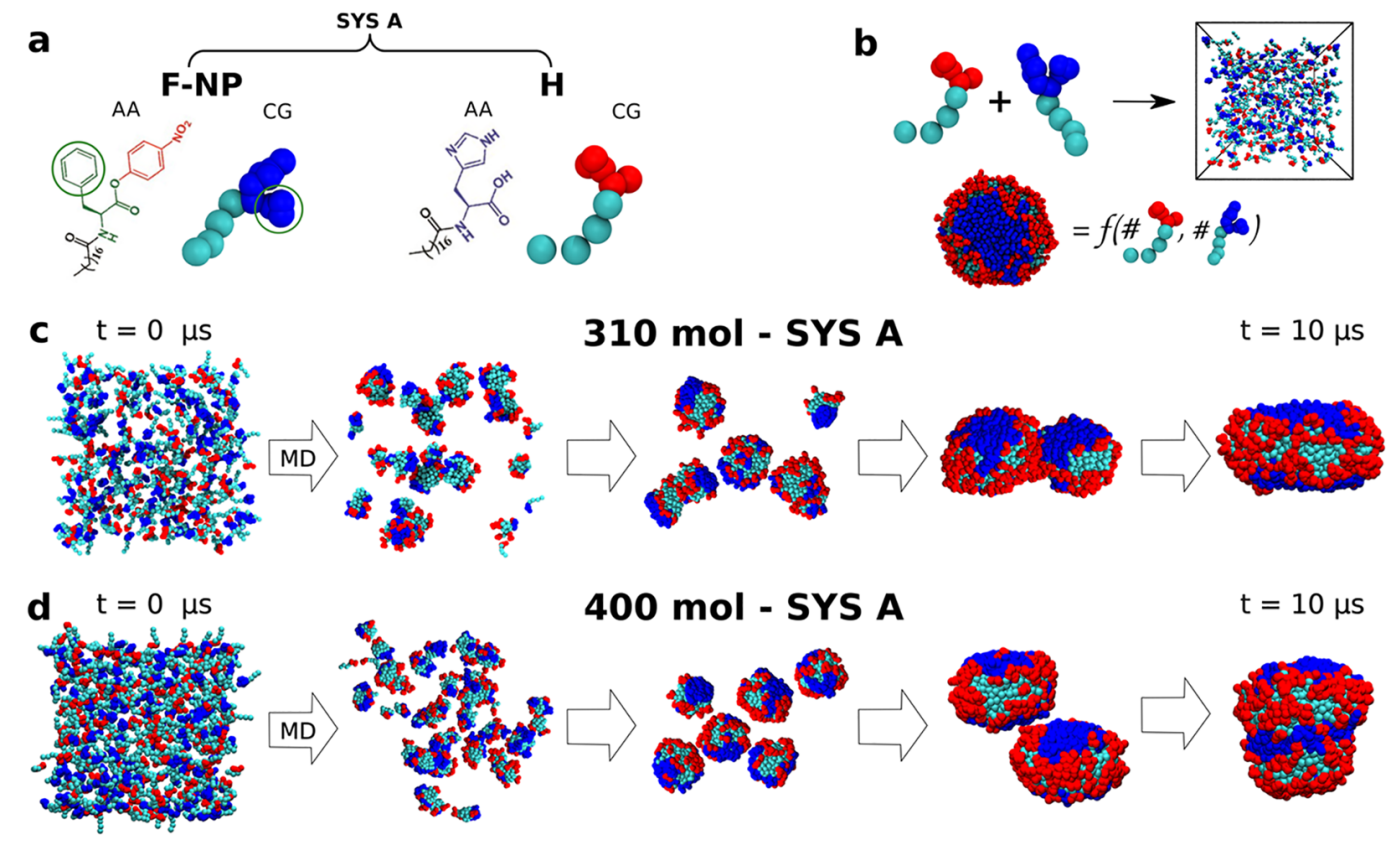
Finer chemically relevant bicomponent micelle models. (a) Chemical structures (AA) and fine coarse-grained (fCG) models of **SYS A** surfactants: *p*-nitrophenyl ester of *n*-stearoyl l-phenylalanine, **F-NP** and *n*-stearoyl l-histidine, **H**. (b) Increased size micelles made of 200, 310, or 400 surfactants, respectively, have been obtained via self-assembly of an equal number of red and blue surfactants during fCG-MD simulations. (c,d) Representative snapshots of 10 μs self-assembly fCG-MD simulations to form two examples of **SYS A** micelles containing 310 (c) and 400 (d) surfactants in total.

All-atom models (AA) of the surfactants of Figure 3a were initially built in Avogadro^42^ and parametrized by using the OPLS-AA force-field.^43^ All van der Waals interactions were modeled using Lennard-Jones potential (LJ), implemented with a cutoff of 1 nm and a standard geometric-mean mixing rule for unlike atoms. The short-range electrostatic interactions were instead evaluated by summing all particle contributions within a cutoff of 1 nm, while for the remaining long-range interactions a Particle-Mesh Ewald (PME) summation was applied in Fourier space.^44^ In order to develop the fCG models, we first solvated each AA biosurfactant in a cubic box of 5 nm filled with explicit SPC/E water molecules;^45^ then, we carried out a production run lasting 10 ns in the *NPT* ensemble.^40^ Considering the AA-MD trajectories as a reference and applying the standard four-to-one mapping in line with the MARTINI force field scheme,^38^ we used the Swarm-CG tool^46^ to automatically optimize the bond, angles, and dihedral distributions of the fCG beads (see Figure S3 in the Supporting Information for CG models validation). Then, the standard MARTINI 2.2 force-field^38^ in explicit water (W) was adopted to describe the nonbonded interactions among the beads. All model parameters are available at https://zenodo.org/record/7696708#.ZAI0A3bMI2w.

#### AA and fCG-MD Simulations

The AA-MD simulations of each surfactant include a total energy minimization and two equilibration steps to achieve *T* = 300 K and *p* = 1 bar: the former in *NVT* ensemble and the latter in *NPT* ensemble for 100 ps using the v-rescale thermostat^47^ (τ_*T*_ = 0.1 ps) and the Parrinello–Rahman barostat^41^ (τ_*p*_ = 2 ps). Once the equilibrium thermodynamic conditions were reached, we carried out additional 5 ns of production run in the *NPT* ensemble by implementing a Nose–Hoover thermostat^48^ (τ_*T*_ = 0.4 ps) and the Parrinello–Rahman barostat^41^ (τ_*p*_ = 8 ps).

The self-assembly CG-MD simulations were carried out following the MARTINI parametrization in explicit solvent.^38^ In particular, no-polarizable-type P4-martini beads have been used to model the explicit solvent, without any ionic strength. The simulation box dimensions are *L*_*x*_ = 20.0 nm, *L*_*y*_ = 20.0 nm, and *L*_*z*_ = 20.0 nm in the *x*-, *y*-, and *z*-directions, respectively, thereby containing a number of water beads ranging from 55000 to 60000 in order to keep a constant pressure while tuning the surfactant concentration. Our simulation protocol consists of a 50.0 ns of equilibration run to thermalize the system at *p* = 1.0 bar and *T* = 300 K; in this step, we used the v-rescale thermostat^40^ (τ_*T*_ = 2 ps) and Berendsen barostat^49^ (τ_*p*_ = 12 ps). During the production runs, lasting 10 μs, we applied the v-rescale thermostat and the Parrinello Rahman barostat,^41^ still maintaining *p* = 1.0 bar and *T* = 300 K. A time step of 20 fs has been used to integrate Newton’s equations of motion. Short-range interactions have been truncated at 1.2 nm. Three-dimensional periodic boundary conditions were applied. All simulations have been performed using the open-source code GROMACS 2018.6.^39^

### Unsupervised Machine Learning

#### SOAP Analysis

In order to study the internal organization and dynamics of micelles, a good representation of the molecular surrounding of each surfactant head along the MD simulation is needed. For this purpose, we parsed the equilibrium MD trajectory of the center of mass (COM) of each surfactant headgroup into a mathematical object, the Smooth Overlap of Atomic Positions (SOAP).^50^ Although a number of descriptors, like atom-centered symmetry functions (ACSFs),^51,52^ and the many body tensor representation (MBT)^53^ have been proposed for designing invariant features of materials, SOAP has demonstrated a remarkable flexibility to describe local environments in diverse materials, from soft to crystalline structures.^9,31^ For every sampled frame along the CG-MD simulation, SOAP describes the local distribution and structural organization of all surfactant head COMs within a specific cutoff radius. It is worth noticing that the resolution of the starting model, either it is atomistic or coarse-grained, and the number of SOAP centers chosen to identify the structural distributions of building blocks is absolutely arbitrary and it is strictly linked to the purpose and the physical-chemical phenomena one is interested in. The equilibrated CG-MD micelle trajectories (last 5 and 3 μs for the mCG and fCG models, respectively) were extracted every 10 ns and analyzed via DScribe,^52^ obtaining a SOAP data set ranging from roughly 60000 to 100000 data points per micelle. The single SOAP data point, corresponding to the *i*th center, is the partial power spectrum vector **p**(**r**), where its elements are defined as
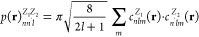
1where *c*_*nlm*_^*Z*^(**r**) are the expansion coefficients of the particle density surrounding the *i*th-center, *n* and *n*′ are indices for the different radial basis functions up to *n*_*max*_, *l* is the angular degree of the spherical harmonics up to *l*_*max*_, and *Z*_1_ and *Z*_2_ are atomic species. Thus, the SOAP output is a high-dimensional vector encoding environmental information in proximity of the considered center of application, within a selected cutoff radius *r*_*cut*_ (see Figure 1b).

#### Dimensionality Reduction and Unsupervised PAMM Clustering

**Figure 4:**
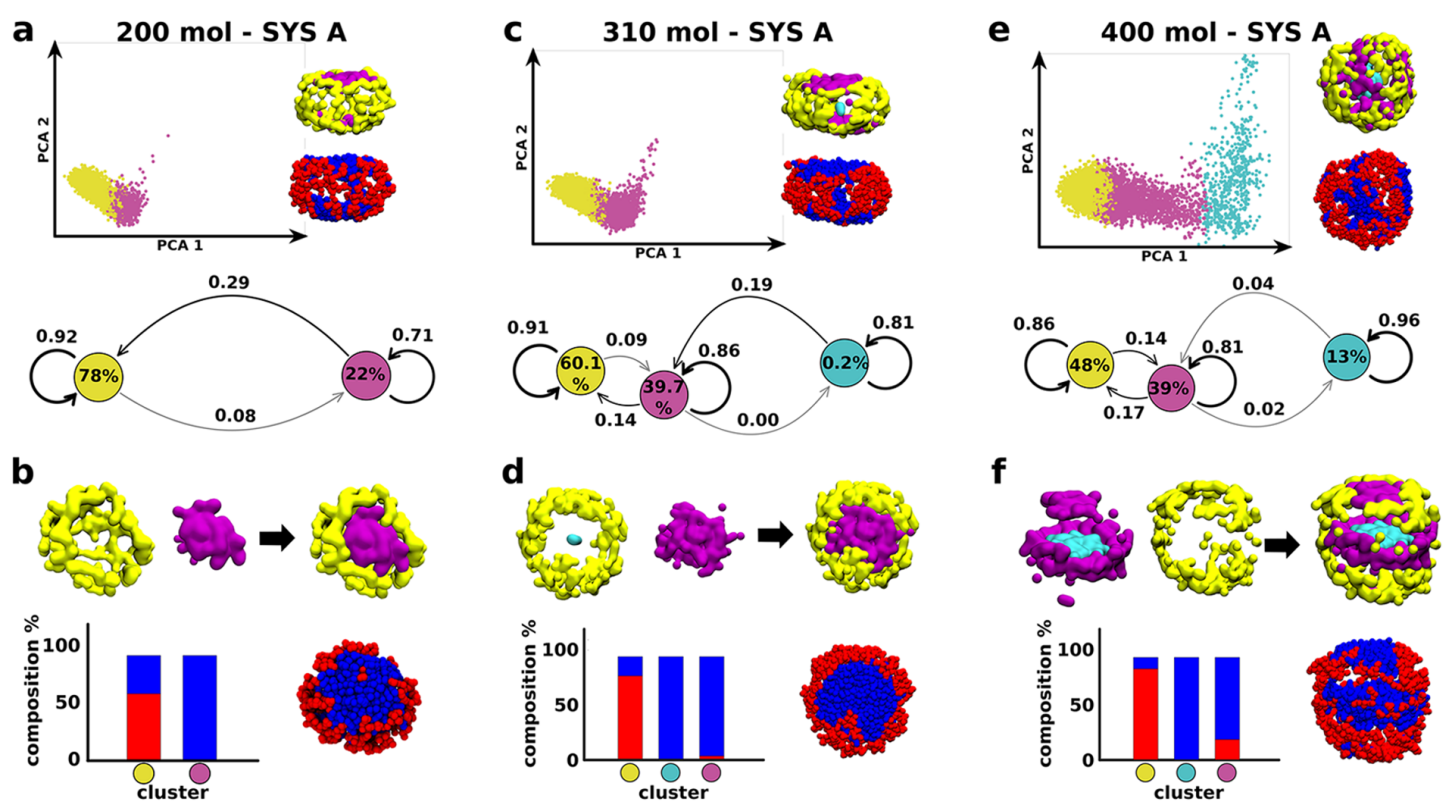
SOAP data set clustering of **SYS A** micelles containing 200 (a,b), 310 (c,d), or 400 (e,f) surfactants in total. (a,c,e) Top: PCA projections of the SOAP data sets on the first two Principal Components with the side view representation of micelles according to both the cluster identification and the molecular species details at fCG-MD time = 10 μs (a,c) and 9.3 μs (e). Bottom: Cluster interconversion diagrams, reporting (i) the surfactant populations per cluster (percentages inside the colored circles), (ii) the probabilities for surfactants to remain within a given cluster (arrows starting/ending from/to the same colored circle), and (iii) the transition probabilities toward a different cluster (arrows connecting diverse colored circles) in the time interval of the analysis (d*t* = 10 ns). (b,d,f) Top: Equilibrium fCG-MD snapshots of micelles at 10 μs showing the decomposition in SOAP-detected clusters. Bottom left: bar plot of the percentage composition per cluster in terms of red and blues amphiphiles. Bottom right: Equilibrium fCG-MD snapshots of micelles at 10 μs showing the molecular species distinction.

Dimensionality reduction of the SOAP data was performed via Principal Component Analysis (PCA), by using TwoNN algorithm^54^ and keeping the *n*th Principal Components (PCs) of the reduced vectors. A number of dimensionality reduction techniques have been used in the analysis of molecular simulations. Principal component analysis (PCA) and Multidimensional scaling (MDS)^55^ are two examples of linear projection methods, while Isomap,^56^ Kernel PCA,^57^ and Diffusion map^58^ are notable for the nonlinear projection method of increasing complex manifolds. Beyond such reduction techniques, other approaches demonstrate that the most relevant degrees of freedom for a system are those in which the dynamics are slower, such as the time-lagged independent component analysis (tICA)^59^ or the spectral gap optimization of the order parameter (SGOOP)^60^ method. Although the above-mentioned techniques offer a valid alternative to reduce the descriptor dimensionality and to explore the dynamics of a complex system, a more traditional and simple approach, like PCA, has been selected for the current work, thereby avoiding greater computational power, a longer trial-and-error procedure, while maintaining a modular design of our computational platform between structural and dynamic analysis. Thus, we decreased the dimensionality of the SOAP spectra from 324 to 5 in order to obtain at least 80% of the total cumulative variance of our data set, as reported in Table 1. We plotted, just for visualization purpose, only the first two PCA components (as in Figures 1d, 2a,b,c, 4a,c,e, left, 6a,e, and 7a,e). The PCA algorithm has been trained on the complete SOAP data set, containing the SOAP vectors corresponding to the micelles that have been compared.^9,31^ Unsupervised clustering of the PCA-reduced SOAP data has been performed using the Probabilistic Analysis of Molecular Motifs (PAMM) clustering algorithm,^31,61^ a density-based clustering method already applied in literature to unveil molecular features in MD systems.^9,10,31^ The PAMM algorithm builds a Probability Distribution Function of the input vectors (the considered PCAs components of the *whole* data set) by the Kernel Density Estimation (KDE) algorithm, on a grid of ngrid points selected through a farthest point sampling method. Then, the Gaussian Mixture Modeling (GMM) clustering algorithm assigns a cluster to each density peak. All the input parameters used to compute the SOAP-based vectors and to apply the PCA and PAMM clustering analyses are detailed in Table 1.

**Table 1:** Parameters Set in the Unsupervised Machine-Learning Analysis^a^

	SOAP		PCA		PAMM	
	cutoff [Å]	*D*	nPC	var [%]	Ngrid	fs
mCG (Figure 1)	30	324	5	80.2	1500	0.1
mCG (Figure 2)	30	324	5	88.5	2500	0.2
fCG (Figure 4)	40	324	5	90.1	200	0.2
fCG (Figure 6)	50	324	5	87.9	3800	0.2
fCG (Figure 7)	50	324	5	87.9	3800	0.2

a Note that D is the SOAP vector dimension computed for the relative cutoff. nPC and var are the number of principal components and variance regarding the dimensionality reduction analysis (PCA), respectively. Ngrid and fs are the number of grid points and a localization parameter of the anisotropic multivariate Gaussian, respectively.

Starting from the clustering analysis data, we defined for each micelle the interconversion diagrams by counting the total number of transitions between clusters, in each frame along the equilibrated CG-MD trajectories. Then, we computed the conditional transition probabilities per micelle by normalizing the results over the total number of transitions initiated from the considered cluster. The population diagrams are instead obtained by averaging the distribution of clusters along the analyzed trajectories. Finally, we checked the composition of each cluster looking at the specific amphiphile species belonging to it.

## Results and Discussion

### Physical Factors Controlling Bicomponent Micelles

We start our investigation by developing a minimalistic coarse-grained (mCG) model of a self-assembled bicomponent micelle allowing us to easily verify the key factors controlling it from a structural and dynamical point of view and to clearly validate our ML-based protocol. In this simple minimalistic model, each surfactant molecule is modeled as a five-bead amphiphile (see Figure 1a): four smaller beads are used to represent the solvophobic flexible surfactant tail and one larger bead to mimic the solvophilic head. The noncovalent interactions between the amphiphile beads are described by a Lennard-Jones (LJ) potential, whose parameters LJ σ and ϵ are specified in Table S1 and Table S2 of the Supporting Information. The interaction matrix between the CG beads is tuned in such a way to have 200 initially disassembled mCG surfactants which self-aggregate, during a classical CG-MD simulation, into a typical micellar structure (i.e., solvophobic tails gathered in interior and solvophilic surfactant heads displaced on the surface of the micelle; see Figure 1a and Figure S1 in the Supporting Information). In particular, in all mCG model variants compared herein, the solvophobic tail beads are kept constant and the tail–tail interactions are thus always the same. Such mCG is an implicit-solvent model: solvent molecules are not explicitly present in the simulation box, and a stochastic dynamics of mCG surfactants implicitly account for the role of the solvent. Additional technical details are provided in the Supporting Information, while complete information on the mCG force field parameters are described in the Methods and in the Supporting Information. It is worth noticing that this mCG model does not aim at describing a particular micelle composed of specific surfactants, but rather at being representative of a general micelle whose assembly feature is controlled by the amphiphilic nature of its building blocks.

Starting from such a minimalistic 200 surfactant micelle model, we generated different types of bicomponent micelles. In particular, we differentiated the 200 self-assembled amphiphiles into two distinct surfactant species, identified in Figure 1b with red and blue colored heads, namely, 100 **R** plus 100 **B** amphiphiles, respectively. The diversity between the two surfactant species is modeled following two distinct approaches: (1) by modulating the homo vs hetero head–head noncovalent interactions or (2) by changing the size of amphiphile heads. Both (1) and (2) approaches mimic to some extent what happens when one changes the headgroups of realistic surfactant molecules, being two distinct molecules in general nonidentical in size and physical-chemical affinity with each other. Although in real systems, altering surfactant species implies most often changing both (1) and (2) simultaneously, in this first phase, we exploit the flexibility of the mCG model to investigate the effects of interaction energy (1) and head size (2) separately, as this offers a clearer picture on key factors dictating either a uniform mixing of the two species or their complete segregation in distinct domains.

As previously mentioned, the micelles of Figure 1b are formed via self-assembly of 100 **R** plus 100 **B** initially dispersed amphiphiles obtained during long CG-MD simulations (see Methods for details). During this simulation time, all micelles reached a dynamic equilibrium with good stability assuming different structures, in which a continuous reshuffling and exchange of surfactants can be observed (Figure 1b). We start our analysis from modulating the homo vs hetero head–head noncovalent interactions, still maintaining the same head bead size. As shown in Figure 1b, we single out three distinct case studies: (left) equal intra- and interspecies pair potential, (center) homointeractions stronger than heterointeractions, (right) diverse homointeractions for the two types of surfactants (blue and red).

To retrace the structural and dynamical complexity of these self-assembled micelles, we turn to a data-driven approach recently used also for other dynamical supramolecular materials.^9,31^ In particular, we use SOAP vectors^50^ as high-dimensional descriptors of the local molecular environments surrounding every surfactant headgroup within a SOAP cutoff (see Methods for details of the analysis). Thus, we obtain a characteristic SOAP spectrum for every single surfactant at each analyzed MD time step, which is indicative of the level of neighboring order/disorder of all surfactant heads on a micelle. SOAP data are collected every 10 ns over the last 5 μs of the equilibrated phase mCG-MD trajectories, for a total of 500 snapshots representative of the equilibrium dynamics of the micelles. We thus obtain a rich SOAP data set (100000 SOAP spectra: 200 SOAP spectra, one for each surfactant, at each of the 500 sampled time steps), allowing us (i) to identify the dominant structural environments on the micelle surface in terms of heads arrangements and ordering and (ii) to track the variability of such environments by considering the monomer reshuffling.^9,31^ For (i), the probabilistic analysis on molecular motifs (PAMM) method^61^ was carried out on the dimensionality-reduced SOAP-data set (see Methods for further details). PAMM algorithm detects three dominant clusters or surfactant head domains on the micelle surface. These are shown in green, purple and orange both on the PCA projection of the SOAP data set (see Figure 1d) and in the equilibrated snapshots in Figure 1f, left. Such SOAP clusters correspond to different micelle regions, each one characterized by a peculiar surfactant structural ordering: the orange heads are gathered into the flatter top and bottom of the micelle (being these micelles not perfectly spherical), while the purple and green heads arrange on a less dense corona.

For (ii), at every sampled MD time step, our analysis keeps track of the specific cluster which every surfactant belongs to. In this way, we are able to reconstruct dynamical information on the exchange probabilities of surfactants to transient from one domain to an other one along the MD simulations, thereby estimating the characteristic transition rates. The interconversion diagram in Figure 1e renders both the percentage of surfactant population per cluster (percentages inside the colored circles) and the conditional transition probabilities (numbers on the transition arrows) for a surfactant to stay in a given cluster or to exchange to another one within the selected time interval (d*t* = 10 ns). The most populated cluster in all studied cases is the purple one, containing ∼49–53% of the surfactants in the micelles. The orange cluster counts ∼38% of the surfactants in all cases. The green state is found more adjacent to the purple one in the PCA (Figure 1d). This is also the least populated cluster (∼9–13% of the surfactants), identifying local surfactant domains within the purple environment. The intracluster transition arrows show that the purple and orange clusters are dynamically more persistent than the green one, with a probability to remain in those environments of ∼55–63% on average. The residence probability is reduced to ∼17–24% in the green cluster, where the surfactants possess a larger mobility. The transition arrows also clarify that the most favorable kinetic pathway for the exchange of surfactants is the one between adjacent clusters—green-to-purple, purple-to-orange—while in all cases the green-to-orange transitions are way more unlikely.

Although such kinetic data are extracted from an approximated CG model and should be thus considered as qualitative, they offer several insights in a comparable analysis after introducing some variants in the description of surfactants. In fact, comparing the distributions of the **R** and **B** amphiphiles in Figure 1b and the cluster reconfiguration in Figure 1f, left, we can correlate the SOAP-detected environments (clusters) to the surfactant identity which compose them. The normalized histograms of Figure 1f confirm such qualitative observation: for the first two case studies, i.e., micelle in Figure 1b, left, and micelle in Figure 1b, center, the SOAP clusters (green, orange and purple) are composed of 50% **R** and 50% **B** amphiphiles. This means that there is not a favorable structural environment where the surfactants would prefer to reside. Either they are completely mixed up (Figure 1b, left) or they are phase separated (Figure 1b, center) we always observe an equal probability to rearrange in a particular cluster. Particularly, in the case study of Figure 1b (center), **R** and **B** amphiphile heads have the same steric hindrance, the **R**–**R** and **B**–**B** interactions are identical, while the cross interaction is weaker. An example of such a case could be, e.g., mixing two amphiphilic molecules where the solvophobic tails are identical, while the heads have the same structure but opposite chirality. In this case, the two amphiphile types clearly segregate in two distinct domains (Figure 1b, center), which do not correspond to structurally different regions on the micelles, as demonstrated by the histograms in Figure 1f, center, where all SOAP clusters are populated 50%–50% by the two amphiphiles. A slightly different prospect is instead outlined in the last case study (Figure 1b, right) where there is a nonuniform distribution of the species in the various SOAP domains. In this sense, it is interesting to investigate the minimum requirements, in such unsupervised data-driven analysis, to classify and distinguish the presence of different molecular types from the trajectories. Here, it is enough to vary one of the homosurfactant interactions to enable a match-up between molecular identity and SOAP-detected environments.

The general nature of these minimalistic physical micelle models offers opportunities to investigate further the factors that control the structural-dynamical behavior of such assemblies. In addition, in light of consistent and formulated classical self-aggregation theories, such a simple mCG model of micelles allows us to unveil and test the robustness of our data-driven analysis. Based on approach (2) mentioned above, we now consider the effect of encoding geometrical features into **R** and **B** surfactants differentiating them.

As an example, we reduce the radius of the **B** surfactant heads compared to that of **R** surfactants, which is kept the same as before (LJ σ_**R**_/σ_**B**_ = 1.49; see Figure 2a–c). The results in Figure 2 show the effect of such structural change. The SOAP analysis on surfactant micelle identifies three main structural clusters, gray, fuchsia, and cyan. The cyan cluster is localized on the bottom and topmost flat regions of micelles; instead, the gray and fuchsia SOAP domains are distributed in the less dense corona all around the micelles (see the mCG-MD micelle snapshots showing the SOAP clusters distribution in Figure 2a–c, center). The interconversion diagrams reveal a reasonable stability of cyan cluster: regardless of the specific case study, the probability of surfactants to remain in the cyan cluster in the time interval d*t* ranges between ∼68 and 88%. The transition of surfactants into cyan environments directly from nonadjactent (on the PCA) gray domains is very unlikely (below ∼3%), while the exchange with the adjacent fuchsia regions is more favorable (∼10–29%). Increased intermixing is observed between gray and fuchsia clusters in all cases.

Introducing the size effect to the tuning of interactions among the surfactants highlights interesting consideration regarding the correlation between the structural environments identified by the data-driven analysis and the surfactant species populating them. Even for LJ ϵ_**RR**_ = ϵ_**BB**_ = ϵ_**RB**_ the distribution of **R** and **B** amphiphiles is not uniform in each SOAP cluster (see histograms in Figure 2a, bottom). In particular, the cyan domains (flatter top and bottom of the micelle) are largely populated by **B** surfactants (∼80%). Although we could expect a mixed up configuration of the species (an energetically favorable rearrangement may have small headgroups, i.e., **B** surfactants, surrounding the largest ones, **R** surfactants, as suggested by the averaged sigma of LJ parameters in Table S2), a kind of phase separation between **B** surfactants and **R** surfactants emerges (see histograms in Figure 2a, bottom). A couple of reasons can be found while explaining such behavior. On one hand, because the **B** surfactants are characterized by the smallest sigma, and thus by the shortest range of interactions, they tend to self-aggregate, rather than surround red surfactant heads; on the other hand, the topological feature of **B** surfactants is such to privilege a double layer reorganization and thus to rearrange in flatter regions on micelle surface, as largely demonstrated by classical molecular thermodynamics theories. The combined effect of these two phenomena may lead to a slight compartmentalization of the two species into separate regions. The system in Figure 2b displays a clear compartmentalization. Here, the cyan and gray clusters effectively include >95% of the **B** or **R** surfactants, respectively. This confirms the outcomes of the interconversion diagrams where the transitions of amphiphiles between the cyan and gray micelle regions are extremely unlikely. Cyan-gray exchange may mainly occur via involving the intermediate fuchsia cluster, which results as more intermixed of **B** or **R** surfactants in all studied cases (Figure 2a–c, bottom). A more enhanced distinction among **B** or **R** surfactants is also shown in Figure 2c.

The case studies shown in Figure 2, although simple and minimalistic, are quite explicative to demonstrate the challenging task to predict the exact configuration and structural rearrangement of surfactants by simply providing the topological, and force field details. In most of the cases, the internal reorganization of soft aggregates is the result of combined effects, and the identification of a predominant behavior is not really straightforward. Such consideration pushed our research toward more sophisticated analyses, which are more and more versatile, flexible, and transferable to a number of diverse soft self-assemblies. In other words, the minimalistic models here presented clarify that such a data-driven ML analysis allows us to accurately unveil the structural and dynamic properties of diverse aggregate, namely, based on how the different molecules arrange, interact, and move with respect to each other, and without any *a priori* information on their species. This has thus considerable potential when used on chemically relevant higher resolution models of realistic molecular systems, where greater chemically detailing is maintained.

### Increasing Complexity in Realistic Bicomponent Micelles

**Figure 5:**
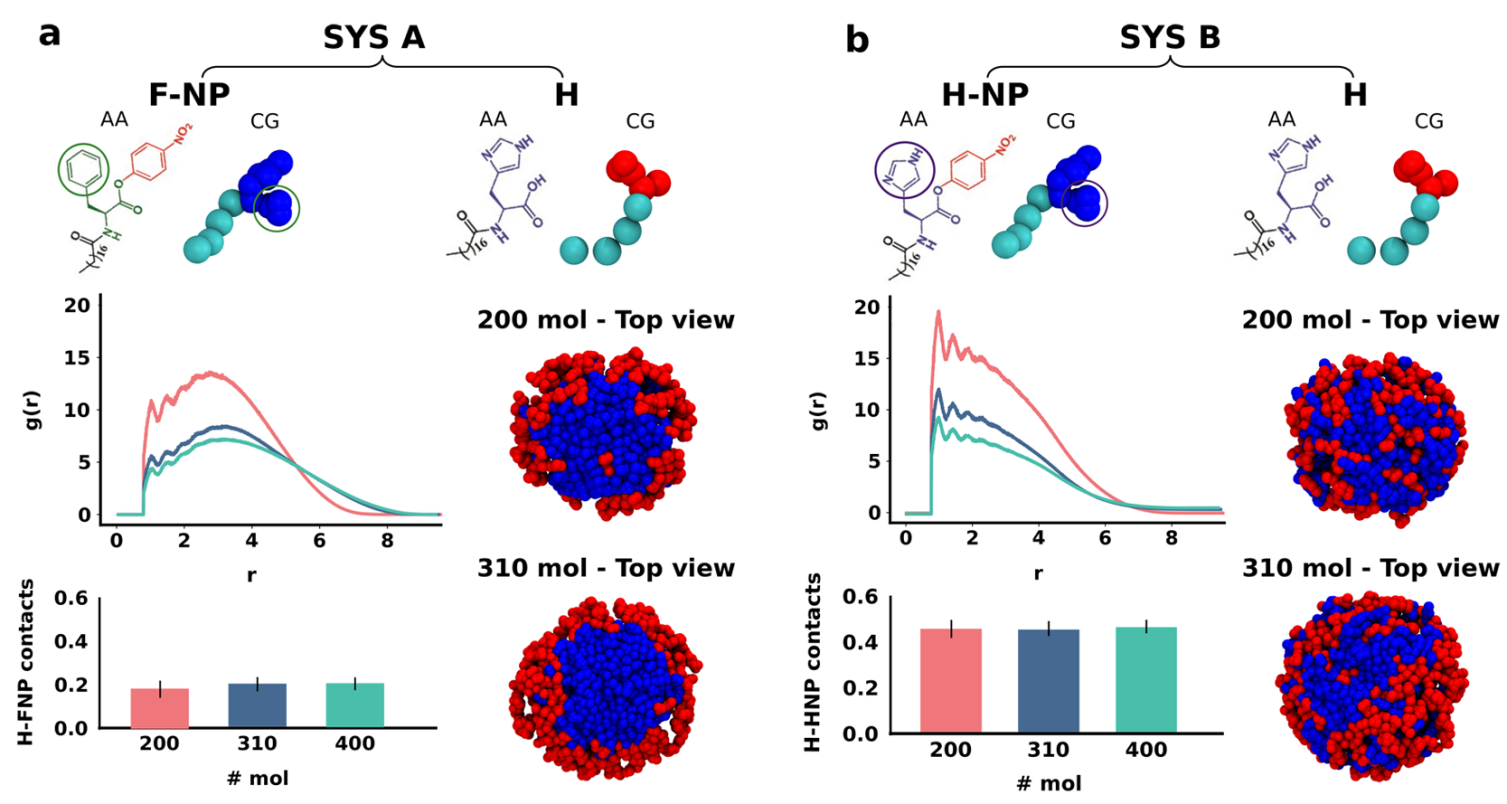
Finer chemically relevant bicomponent micelle models of both **SYS A** and **SYS B** systems. (a,b) Top: Chemical structures (AA) and fine coarse-grained (CG) models of *p*-nitrophenyl ester of *n*-stearoyl l-phenylalanine, **F-NP** (a), *n*-stearoyl l-histidine, **H** (a,b), and *p*-nitrophenyl ester of *n*-stearoyl l-histidine **H-NP** (b). Although the same color code, **F-NP** and **H-NP** have distinct noncovalent parameters for those beads mapping chemically diverse rings highlighted in green and purple circle. (a,b) Center: Radial distribution function, *g*(*r*), between the hydrophilic heads of **H** monomer and the center of mass of hydrophobic tails representing the micelle core. Pink, blue, and cyan *g*(*r*) profiles correspond to 200, 310, and 400 surfactant micelles, respectively. (a,b) Bottom: Average number of contacts per single couple, **FNP**-**H** or **HNP**-**H**, in **SYS A** (a) or **SYS B** (b), respectively. Note that the contact count is carried out only considering surfactant head beads.

As realistic molecular examples, we use recently reported bicomponent micelles formed via self-assembly of *n*-stearoyl l-histidine (**H**) with either *p*-nitrophenyl ester of *n*-stearoyl l-phenylalanine (**F-NP**) or *p*-nitrophenyl ester of *n*-stearoyl l-histidine (**H-NP**) amphiphiles.^13^ As follows, we label as **SYS A** and **SYS B** the previous two cases, respectively (see Figure 5a,b). We start focusing our investigation on **SYS A**. As shown experimentally, the self-assembly of **F-NP** and **H** molecules allows some interesting structural configurations, which in the real system enable and accelerate eventually catalytic reactions between different surfactants at specific concentrations.^13,14^

We adopt a finer coarse-grained (fCG) scheme based on the widely used Martini force field,^38,62^ which was proven reliable for studying self-assembling systems and for preserving most of the chemical details of the modeled molecules.^9,63^ The fCG models of the amphiphiles involved in **SYS A** are shown in Figure 3a where red and blue colors are selected to distinguish the head beads belonging to diverse surfactant species within the same micelle. As displayed in Figure 3b,c,d, the MD simulations start from a well dispersed solution in explicit water (see Methods for water model details) containing an equal amount of **H** and **F-NP** amphiphiles. We set up three different solutions of increasing concentration, made of 200, 310, or 400 surfactants in total. 10 μs of CG-MD self-assembly simulations were then carried out to obtain three equilibrated micelles of increasing size, while the data analysis was performed on the last 3 μs of the equilibrium phase trajectories. Representative snapshots of the CG-MD self-assembly simulations are reported in Figure 3c,d showing the final shape at *t* = 10 μs of 310 and 400 surfactant micelles, respectively. As clear from the last two snapshots of each CG-MD trajectory, the final rearrangements of amphiphiles are strongly affected by the nature and the orientation of smaller micelle aggregation, determining different structural reconfigurations of amphiphiles and diverse pathways of self-assembly. It is worth noticing that we work in surfactant concentration conditions well above the Critical Micelle Concentration (CMC);^13^ consequently, all amphiphiles likely self-assemble and the probability to observe some surfactants in monomeric form within the solution is very low. In addition, larger time and space scales would be required to capture such extra phenomena.

The SOAP-based clustering analysis on the considered three micelles, i.e., those one made of 200, 310, or 400 surfactants belonging to **SYS A**, was conducted following the same protocol presented for the minimalistic models in Figures 1 and 2. As reported in Figure 4, the SOAP and PAMM combined analysis identifies three main clusters, colored in yellow, magenta, and light blue in Figure 4c–e. A quantitative signal of the structural diversity between the three different-sized micelles is captured and reflected in Figure 4a,c,e by the PCA projection of the SOAP data and by the interconversion diagrams. The yellow cluster is the most populated, with a percentage of surfactants ranging from ∼48 to ∼79%. Regardless of the micelle size, such a yellow environment mostly identifies the corona-like region. On the other hand, the light-blue cluster corresponds to an internal domain, weakly populated (∼0.2% and ∼13% of the amphiphiles in the case of 310 and 400 molecules, respectively), and never at the water interface. As shown in the intermediate size (see Figure 4c,d) and in the largest size micelles (see Figure 4e,f), the surfactants belonging to the light blue cluster are encapsulated within the micellar aggregate in a kind of double-layer rearrangement. The corresponding PCA and interconversion plots show that such a small cluster identifies a distinct state but yet interconnected to the magenta domain. The percentage of surfactants in such internal light-blue environment drops to only ∼0.2% in the intermediate-size micelle, while this cluster is completely absent in the smallest micelle. Especially in the 310 surfactant micelles, the magenta domain corresponds again to the flatter top and bottom regions of slightly compressed micelles (Figure 4a–d).

The interconversion diagrams in Figure 4a,c,e (bottom) show that all detected SOAP clusters are quite dynamically persistent, with a residence probability (in the time interval, d*t* = 10 ns) > 71%. The unique exception is the magenta cluster in the smallest micelle (Figure 4a,b). Consistently, the surfactant transitions among diverse environments are quite infrequent, or even hindered, as between the yellow and light-blue clusters. This result asserts a reduced mobility of surfactants which substantially preserve their local surrounding once they reach the equilibrium configuration within a micelle. From a purely technical point of view, a higher staticity is expected in these finer-CG models compared to the mCG models, where the sampling is accelerated. Nonetheless, while the transition probabilities/rates should be considered as qualitative as in the mCG, also these finer models prove a considerable internal dynamic complexity. A first qualitative assessing of Figure 4b,d,f shows a striking correlation between the physically/structurally SOAP environments and their composition in terms of surfactant populations (compare micelle snapshots in Figure 4b,d,f). The composition histograms confirm this quantitatively. In particular, the yellow and magenta clusters are mainly composed of **H** (red) and **F-NP** (blue) surfactants, respectively, with limited second-species infiltration due to the non-negligible transferring probability. The light-blue cluster (in the interior of the bigger micelles) is instead completely composed of **F-NP** amphiphiles. This suggests a peculiar structural reconfiguration in these larger micelles, probably related to the chemical structure of the blue heads (containing two aromatic rings), which are eventually encapsulated within the micelle core over a certain concentration. Merging the information from the interconversion diagrams with those of the population histograms, we observe that, in general, a transfer of **F-NP** (blue) surfactants from the light-blue to the yellow clusters is possible only via intermediate transition involving the magenta domains, which work as transfer bridges. Nonetheless, the transition in the other direction, namely, toward the light-blue domain is found to be extremely unlikely.

In order to obtain a more general picture, we also consider an additional case study, **SYS B**, where the diversity between **R** and **B** surfactants is reduced (Figure 5a,b). This produces, in **SYS B** compared to **SYS A**, an enhancement of the interspecies **R**–**B** amphiphile interactions, with a more pronounced internal reshuffling of surfactants. Bicomponent micelles composed of 50% **H-NP** plus 50% **H** (see Figure 5b) were obtained and simulated as done for **SYS A**. A comparative preliminary analysis, correlating micelles of **SYS A** and **SYS B**, is reported in Figure 5a,b, where both radial distribution functions (*g*(*r*)) and contact analysis highlight a substantially compartmentalized or mixed surfactant arrangement in **SYS A** or **SYS B**, respectively. In particular, the radial distribution functions are computed between the hydrophilic heads of the **H** monomer and the center of mass of hydrophobic tails representing the micelle core. In **SYS A** micelles, the *g*(*r*) values demonstrate a higher probability of finding red (**H**) surfactant heads at roughly *r* = 3.5 nm from the micelle core; namely, the *g*(*r*) profiles show a more localized spatial distribution on the corona region of the considered micelles whose gyration radius, indeed, ranges from 3 to 4 nm, as demonstrated in Figure S4. On the other hand, the *g*(*r*) values in **SYS B** micelles highlight a more distributed rearrangement of red (**H**) surfactant heads which are detected both on the external corona region (roughly at *r* = 3.5 nm) and on the top and bottom-most domains (1 nm < *r* < 2 nm) of flattened micelles. The bar plots in Figure 5a,b report the average number of contacts per single surfactant couple, namely, **FNP**-**H** in **SYS A** (a) or **HNP**-**H** in **SYS B** (b). The higher number of contacts between **R** and **B** surfactants in **SYS B** micelles show a more mixed arrangement among the two surfactant species. This is qualitative demonstrated by also comparing the MD snapshots of micelles in Figure 5a,b.

**Figure 6:**
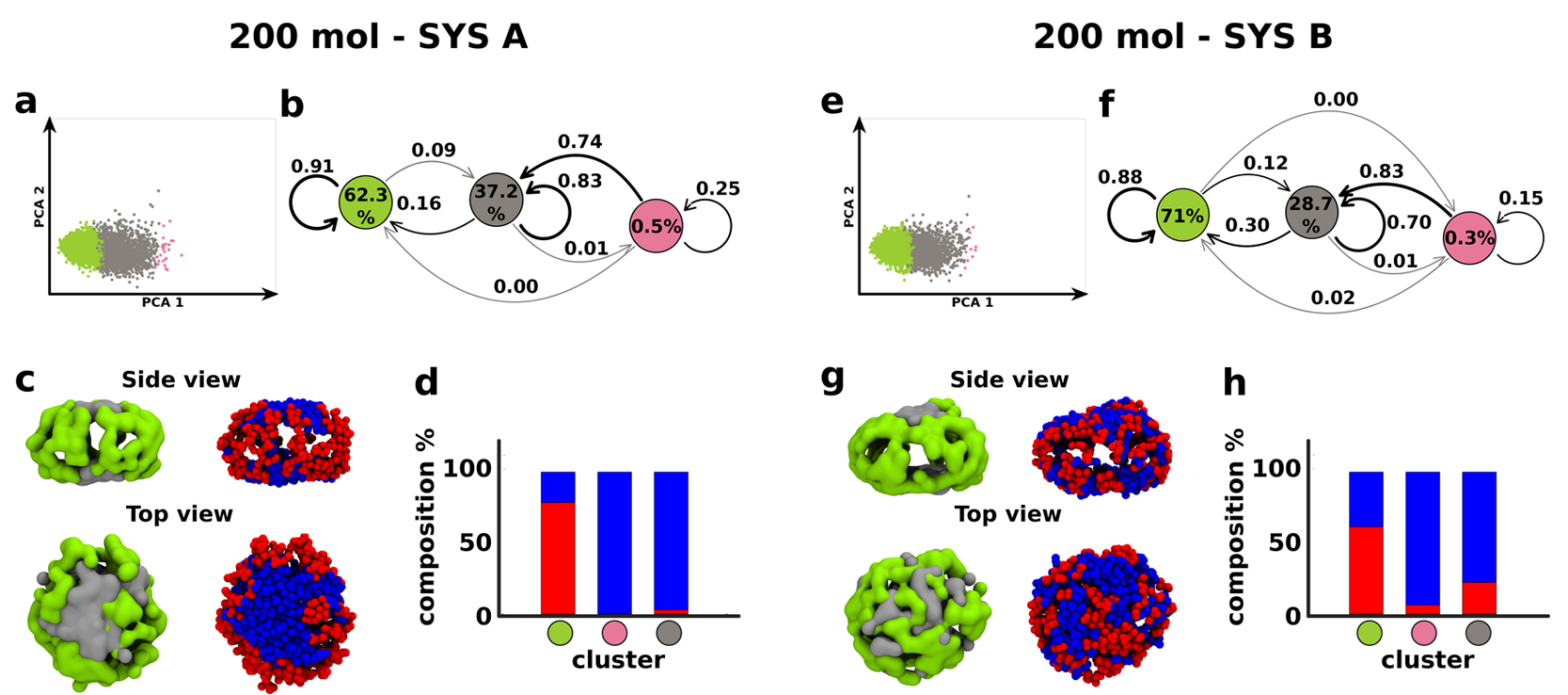
Effect of chemical diversity on the structural and dynamical features of bicomponent micelles. Data obtained from equilibrated fCG-MD simulations of 200 amphiphile micelles in the case of **SYS A** (left) and **SYS B** (right) systems. (a,e): PCA projections of the SOAP data sets on the first two Principal Components. (b,f) Cluster interconversion diagrams, reporting (i) the surfactant populations per cluster (percentages inside the colored circles), (ii) the probabilities for surfactants to remain within a given cluster (arrows starting/ending from/to the same colored circle), or (iii) the transition probabilities toward a different cluster (arrows connecting diverse colored circles) in the time interval of the analysis (d*t* = 10 ns). (c,g) Equilibrium fCG-MD snapshots showing the SOAP-detected clusters (left) and the distribution of red and blue surfactant heads (right) in the micelles. (d,h) Population histograms showing the surfactant composition in each detected SOAP cluster in terms of red and blues amphiphiles.

**Figure 7:**
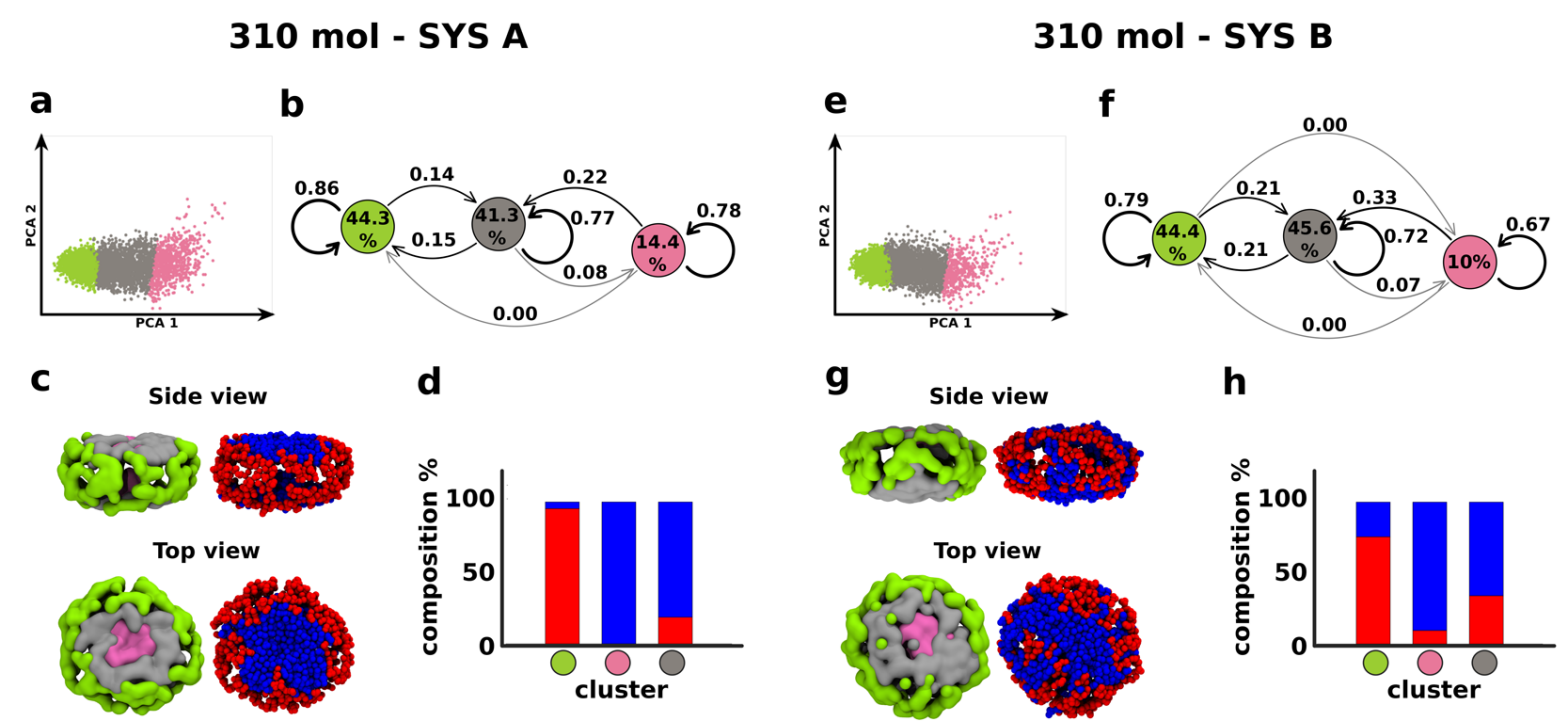
Effect of chemical diversity on the structural and dynamical features of bicomponent micelles. Data obtained from equilibrated fCG-MD simulations of 310 amphiphile micelles in the case of **SYS A** (left) and **SYS B** (right) systems. (a,e) PCA projections of the SOAP data sets on the first two Principal Components. (b,f) Cluster interconversion diagrams, reporting (i) the surfactant populations per cluster (percentages inside the colored circles), (ii) the probabilities for surfactants to remain within a given cluster (arrows starting/ending from/to the same colored circle), or (iii) the transition probabilities toward a different cluster (arrows connecting diverse colored circles) in the time interval of the analysis (d*t* = 10 ns). (c,g) Equilibrium fCG-MD snapshots showing the SOAP-detected clusters (left) and the distribution of red and blue surfactant heads (right) in the micelles. (d,h) Population histograms showing the surfactant composition in each detected SOAP cluster in terms of red and blues amphiphiles.

To provide more quantitative insights, we report the results of our SOAP-based ML study which directly correlates **SYS A** and **SYS B** micelles composed of 200 (Figure 6) and 310 (Figure 7) surfactants. The equilibrium MD trajectories of the four micelles in Figure 5 constitute our sample of analysis. In both **SYS A** and **SYS B** systems, the PAMM unsupervised classification identifies three main clusters: green, gray, and pink, from the most to the less populated respectively, as proven by the population percentages inside the colored circles of the transition diagrams (see Figure 6b,f and Figure 7b,f). In addition, the data show that the larger is the micelle, the more extended becomes the pink environment: the population percentage reaches ∼10–15% in the 310 molecule micelles (Figure 7), while it is just ∼0.3–0.5% in both 200 molecule micelles (Figure 6). A common feature between the two bicomponent micelles of **SYS A** and **SYS B**, respectively, lies in the lack of direct communication between the pink and green clusters, demonstrated by the ∼0% transition probability (see Figures 6 and 7). This indicates the presence of distinct domains in the micelles, which are not interconnected with each other (nor spatially or dynamically). In fact, the pink cluster is seen in the topmost region, surrounded by a gray domain which separates it from the green corona (side and top views of the micelles of Figure 7c,g).

On the other hand, some differences between **SYS A** and **SYS B** systems emerge from a deeper look of the data. Looking at the transition probabilities in the interconversion diagrams, the probability to stay in a given cluster is in general lower in **SYS B** than in **SYS A**. On the contrary, the mobility of surfactants among diverse micelle environments is higher in **SYS B** than in **SYS A**. This provides a quantitative confirmation of the higher surfactant reshuffling preserved in **SYS B** and of the more dynamic character of such bicomponent micelles. The population histograms in Figures 6d,h and 7d,h also remark that, in the **SYS A** system, all detected SOAP clusters are essentially composed of one single surfactant species. In particular, ∼80–95% of the green corona in **SYS A** micelles is composed of red surfactants; on the other hand, the gray and pink flatter environments are filled almost entirely by blue surfactants. In comparison, all SOAP clusters in **SYS B** are more heterogeneous. For instance, the composition of a green cluster in the 200 molecule **SYS B** includes ∼60% and ∼40% red and blue surfactants, respectively. The gray environment in the 310 molecule **SYS B** micelle is composed of ∼70% blue surfactants and ∼30% red ones. It is interesting to note that the clustering process is always relative to the specific data set within which the classification is carried out. This is the reason why some weak differences in the clustering may be identified by comparing the molecular motives of the same micelle but may be included in various starting data sets (see Figures 4, 6, and 7). In other words, the identification of internal structural domains is not univocal, in absolute terms, but it is always in relation to an ensemble of assemblies (data set).

In summary, also in these chemically relevant micelle models we demonstrate that our ML-based analysis is able to unveil the one-to-one correlation between physically/structurally different clusters and their composition in terms of surfactant species. Collecting the results in Figures 4, 6, and 7 with those obtained for the minimalistic mCG models of Figures 1 and 2, we remark the key factors controlling the structural and dynamic complexity of amphiphile micelles. Both the topological differences between the surfactant molecules and the interspecies interactions are found to mostly contribute to the self-rearrangement of surfactants which leads to either a compartmentalization or a complete mixing within a micelle. Beyond such qualitative insights, widely recognized and well-known, our SOAP-based ML analysis provides quantitative insights by reconstructing collective structural motives and uncovering dominant dynamic pathways in terms of transient/residence probability among diverse environments in a number of bicomponent aggregates.

Nonetheless, realistic molecular systems (Figures 4, 6, and 7) provide finer evidence even for slight molecular changes among the two species. This highlights the key role of both structural and energetic features of the building blocks in dictating to what extent they will intermix or segregate in such assemblies. Even taken alone, tiny differences among the amphiphiles induce diverse molecular behaviors in the assembly, and our unsupervised SOAP-based ML analysis is able to capture such perturbation.

## Conclusion

Understanding the structural and dynamic complexity of multicomponent self-assembling systems is not easy. Here we report an unsupervised machine-learning approach to investigate the structural and dynamic behavior of bicomponent micelle models. By coupling high-dimensional SOAP descriptors and unsupervised clustering (PAMM) and by combining finer chemically relevant and minimalistic physical models, we investigate the fundamental factors controlling the self-rearrangement of surfactants in dynamic self-assembled micelles.

The unsupervised ML approach we use here is found to be perfectly suitable to reconstruct the structural and dynamical features of multicomponent micelles. Assembled domains with conformational differences are easily detected by the analysis (e.g., flat and compact vs toroidal less dense domains in slightly compressed micelles). In fact, such a ML-based approach enables us to identify dominant structural environments on micelles, to estimate their stability, and to resolve the dynamic exchange of molecular building blocks among the identified clusters. This provides a comprehensive picture of such micelles including their structural diversity, their dynamic reconfigurability, and the pathways for exchange/reshuffling of self-assembling molecules within them.

In addition to the clustering detection, we also tested the sensitivity of the proposed unsupervised analysis to correlate structural motives with different molecular species simply based on how these arrange and move within the self-assembled micelle. Our results indicate that the formation of structural domains (clusters) in a micelle, characterized by different physical features (flat vs less-dense domains, single vs double layer arrangements), does not necessarily correlate with a segregation of the self-assembling molecular species. Rather, these may be simply due to how the building blocks aggregate in given conditions. Even in such structurally nonuniform micelles, the surfactants can intermix almost completely in all regions of the micelle, provided that they are similar/prone enough to cross-interact. On the other hand, distinct amphiphile species tend to segregate in different micelle environments as far as the coassembled species differ from both topological and interactions points of view. In this sense, the comparison between fCG and mCG models provides a clear evidence of the prime role played by the geometrical features and details of the molecular building blocks. In one, molecular structure encodes in the building blocks different shape–shape recognitions and different intermolecular interactions.

Overall, the unsupervised data-driven analysis approach we report herein stands out as a high-potential platform to reconstruct and understand the structural/dynamical complexity of soft self-assembled micelles, as well as to explore the key factors that may allow us to control their complex behavior.

## Data Availability

Complete details of all molecular models used for the simulations, and of the simulation parameters (input files, etc.) are available at https://zenodo.org/record/7696708#.ZAI0A3bMI2w (DOI: 10.5281/zenodo.7696708).

## Supporting Information Available

Radial distribution functions, LJ parameters, cluster population percentage over time, validation of the bond and angle distributions, and characterization of both systems **SYS A** and **SYS B** self-assembled micelles (PDF)
