# Supplementary material for: Unsupervised Data-Driven Reconstruction of Molecular Motifs in Simple to Complex Dynamic Micelles
Source: J Phys Chem B. 2023 Mar 9;127(11):2595–608. doi: 10.1021/acs.jpcb.2c08726 (PMC10041528; doi:10.1021/acs.jpcb.2c08726)
Supplement: Supplementary file 1 — jp2c08726_si_001.pdf [file jp2c08726_si_001.pdf]

Supplementary Information: Unsupervised Data-Driven Reconstruction of Molecular Motifs in Simple to Complex Dynamic Micelles

Annalisa Cardellini[1], Martina Crippa[2], Chiara Lionello[2], Syed Pavel Afrose[3], Dibyendu Das[3], and Giovanni M. Pavan[1,2,*]

[1]Department of Innovative Technologies, University of Applied Sciences and Arts of Southern Switzerland, Polo Universitario Lugano, Campus Est, Via la Santa 1, 6962 Lugano-Viganello, Switzerland
[2]Department of Applied Science and Technology, Politecnico di Torino, Corso Duca degli Abruzzi 24, 10129 Torino, Italy
[3]Department of Chemical Sciences and Centre for Advanced Functional Materials, Indian Institute of Science Education and Research (IISER) Kolkata, Mohanpur 741246, India
[*]corresponding author: Giovanni M. Pavan (giovanni.pavan@polito.it)

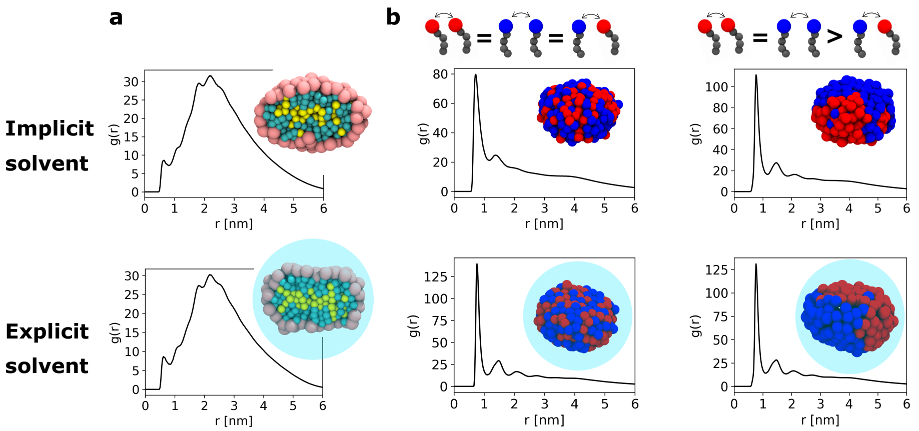

Figure S1: (a) Radial distribution functions (g(r)) of the head beads (pink) respect to the geometric center of the last tail beads (yellow), both in implicit (top) and explicit (bottom) solvent model. (b) Radial distribution functions (g(r)) of the heads distribution around the core of each micelle in case of completely mixed (left) and compartmentalization (right) regime among red and blue surfactants. g(r)s are compared in implicit (top) and explicit (bottom) solvent models.

L-J Parameters	SYSTEM	**R-R**	**B-B**	**R-B**	**R-tail**	**B-tail**	tail-tail
σ[nm]	All	0.7	0.7	0.7	0.585	0.585	0.47
ϵ[kJ mol^{-1}]	Mixing	0.5	0.5	0.5	0.5	0.5	5
	Compartment	4	4	0.5	0.5	0.5	5
	Intermediate	4	0.5	0.5	0.5	0.5	5

Table S1: Non-bonded Lennard-Jones parameters for the **R** and **B** surfactants considered in Figure 1b, in case of mixing (Figure 1b left), compartmentalization (Figure 1b middle) and an intermediate behavior (Figure 1b right).

L-J Parameters	SYSTEM	**R-R**	**B-B**	**R-B**	**R-tail**	**B-tail**	tail-tail
σ[nm]	All	0.7	0.47	0.585	0.585	0.47	0.47
ϵ[kJ mol^{-1}]	Mixing	0.5	0.5	0.5	0.5	0.5	5
	Compartment	4	4	0.5	0.5	0.5	5
	Intermediate	4	0.5	0.5	0.5	0.5	5

Table S2: Non-bonded Lennard-Jones parameters for the **R** and **B** surfactants considered in Figure 2, in case of mixing (Figure 2a top), compartmentalization (Figure 2b top) and an intermediate behavior (Figure 2c top).

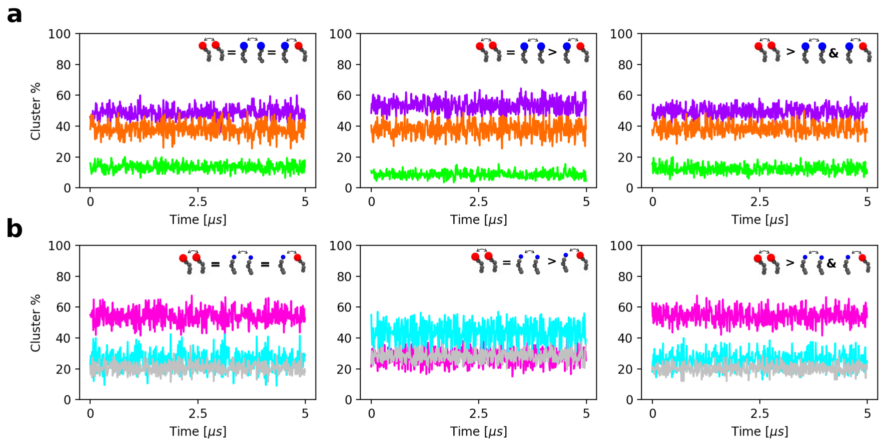

Figure S2: (a) Cluster population percentage over time in case of completely mixed (left), compartmentalization (middle) and intermediate (right) regime for the cases considered in Figure 1. (b) Cluster population percentage over time in case of completely mixed (left), compartmentalization (middle) and intermediate (right) regime for the cases, considered in Figure 2.

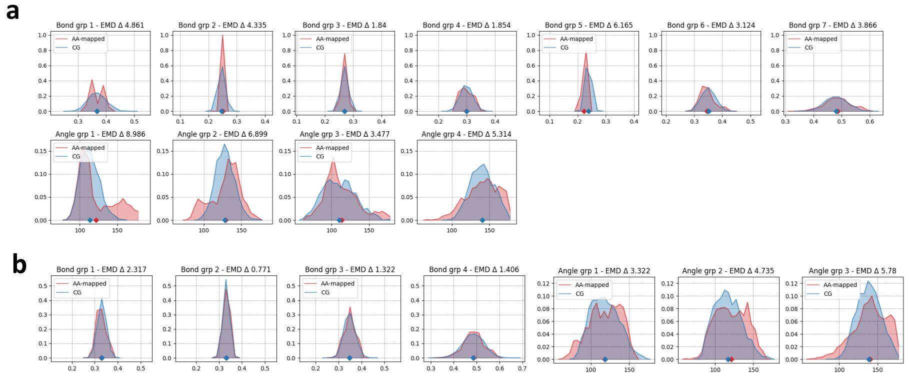

Figure S3: (a) Validation of the bond and angle distributions in a single **F-NP** molecule simulated in both all-atom (AA) and coarse-grained (CG) models by using SwarmCG. (b) Validation of the bond and angle distributions in a single **H** molecule simulated in both all-atom (AA) and coarse-grained (CG) models by using SwarmCG. Note that the model of **H-NP** differs from **F-NP** only for the bead type.

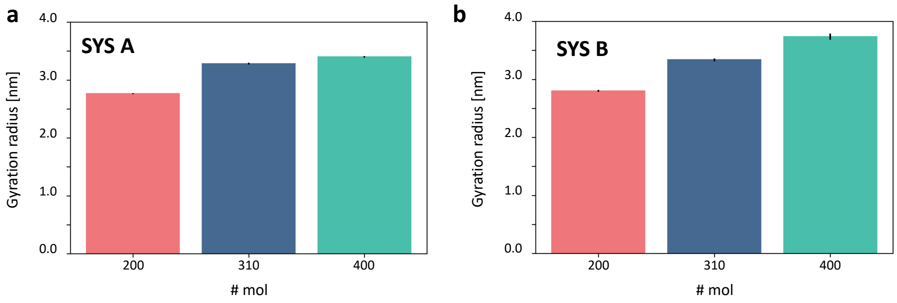

Figure S4: Characterization of both systems **SYS A** and **SYS B** self-assembled micelles. (a-b) Gyration radius of the three different micelles simulated with the fCG model, both in **SYS A** (a) and **SYS B** (b). It can be considered as a reference of the NP dimension.
